# Suppression of La Antigen Exerts Potential Antiviral Effects against Hepatitis A Virus

**DOI:** 10.1371/journal.pone.0101993

**Published:** 2014-07-07

**Authors:** Xia Jiang, Tatsuo Kanda, Shuang Wu, Shingo Nakamoto, Kengo Saito, Hiroshi Shirasawa, Tomoko Kiyohara, Koji Ishii, Takaji Wakita, Hiroaki Okamoto, Osamu Yokosuka

**Affiliations:** 1 Department of Gastroenterology and Nephrology, Chiba University, Graduate School of Medicine, Chiba, Japan; 2 Department of Molecular Virology, Chiba University, Graduate School of Medicine, Chiba, Japan; 3 Department of Virology II, National Institute of Infectious Diseases, Musashimurayama, Japan; 4 Division of Virology, Department of Infection and Immunity, Jichi Medical University School of Medicine, Shimotsuke, Japan; Saint Louis University, United States of America

## Abstract

**Background:**

Despite the development and availability of hepatitis A virus (HAV) vaccine, HAV infection is still a major cause of acute hepatitis that occasionally leads to fatal liver disease. HAV internal ribosomal entry-site (IRES) is one of the attractive targets of antiviral agents against HAV. The aim of the present study is to evaluate the impact of La, one of the cellular proteins, on HAV IRES-mediated translation and HAV replication.

**Methods and Findings:**

We investigated the therapeutic feasibility of siRNAs specific for cellular cofactors for HAV IRES-mediated translation in cell culture. It was revealed that siRNA against La could inhibit HAV IRES activities as well as HAV subgenomic replication. We also found that the Janus kinase (JAK) inhibitors SD-1029 and AG490, which reduce La expression, could inhibit HAV IRES activities as well as HAV replication.

**Conclusions:**

Inhibition of La by siRNAs and chemical agents could lead to the efficient inhibition of HAV IRES-mediated translation and HAV replication in cell culture models. La might play important roles in HAV replication and is being exploited as one of the therapeutic targets of host-targeting antivirals.

## Introduction

Hepatitis A virus (HAV) is a non-enveloped single-stranded RNA virus, with ∼7.6 kb positive-sense genome. The genome includes 5′ non-translated region (5′NTR), one open reading frame encoding structural (VP4, VP2, VP3, VP4 and 2A) and non-structural proteins (2B, 2C, 3A, 3B, 3C and 3D), and 3′NTR [1]. HAV genome translation could be initiated by cap-independent mechanism through HAV internal ribosomal entry-site (IRES) with a pyrimidine-rich tract, which is located at the down-stream part of 5′NTR [2]. HAV is still a major cause of acute hepatitis [3], [4]. Although acute liver failure due to HAV is not common, it is still occasionally fatal [5], despite HAV vaccine having become available [6]–[8]. This emphasizes the importance of the development of antiviral agents against HAV.

In general, two distinct classes of antiviral agents, direct-acting antivirals (DAAs) and host-targeting antivirals (HTAs), exist [9]. Several groups have reported DAAs against HAV, such as inhibitors of HAV 3C cysteine proteinase, which is essential for viral replication and infectivity [10]–[15]. Small interfering RNAs against HAV genome are also varieties of DAAs [16]–[18]. Several broad-target HTAs, examples of which include interferon-α, interferon-β, interferon-λ1 and amantadine, have been developed and tested against HAV [2], [19]–[25]. These compounds could inhibit HAV IRES-dependent translation as well as HAV replication [2], [21], [22]. HTAs of the targeted group are more precise in that they act on key host enzymes or cellular factors that are required for the viral lifecycle [9].

Our previous studies suggested that several siRNAs against HAV 5′NTR suppress HAV translation as well as HAV replication [17]. The nucleotide sequences of 5′NTR are one of the most conserved in HAV genomes [8], [26]. These facts suggest that HAV IRES is one of the attractive targets of antiviral agents against HAV. It has been reported that several cellular proteins such as autoantigen La [27], glyceraldehyde-3-phosphate dehydrogenase (GAPDH) [28], [29], polypyrimidine tract-binding protein (PTB/hnRNPI) [29]–[31], poly(C) binding protein 2 (PCBP2/hnRNP-E2) [32], polyadenylate-binding protein-1 (PABP) [33], eukaryotic translation initiation factor 4E (eIF4E) [34] and eukaryotic translation initiation factor 4E (eIF4G) [33], [35], [36] could interact with HAV IRES *in vitro* or *in vivo*, and could be associated with HAV replication.

Human La protein is predominantly localized in the nucleus and is associated with RNA metabolism [37]. It has been reported that La was associated with U1 RNA [38], telomerase RNA [39], 5′NTR of poliovirus [27], hepatitis C virus (HCV) [40] and GRP78/Bip [41]. La could interfere with IRES-mediated translation.

In the present study, we investigated the therapeutic feasibility of siRNAs specific for these putative cellular cofactors for HAV IRES-mediated translation. It was revealed that siRNA against La (siRNA-La) could inhibit HAV IRES activities as well as HAV subgenomic replication. We also found that JAK inhibitors SD-1029 and AG490, which inhibit La expression, could inhibit HAV IRES activities as well as HAV replication. The present study demonstrated the proof-of-concept for the inhibition of La as a method for suppressing HAV replication.

## Results

### Effects of silencing of cellular factors on HAV IRES-mediated translation

**Figure 1:**
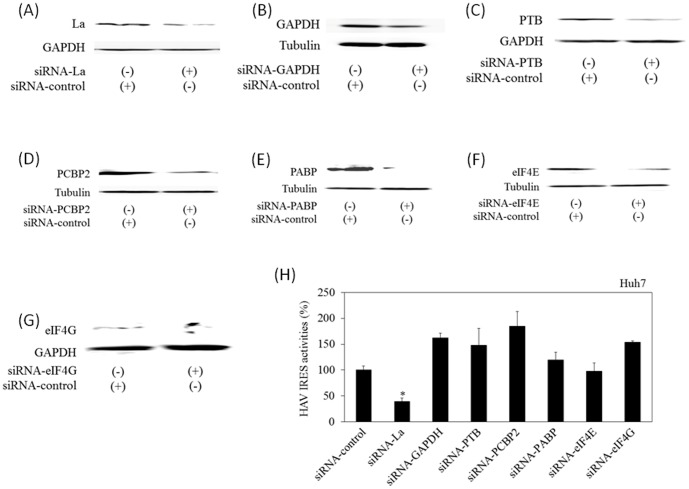
Knockdown of La inhibits hepatitis A virus (HAV) internal ribosomal entry site (IRES) activities. Effects of siRNAs on endogenous gene expression in Huh7 (A–G). Approximately 0.5×10^6^ cells were transfected with 100 nM siRNA against La (siRNA-La), glyceraldehyde-3-phosphate dehydrogenase (GAPDH) (siRNA-GAPDH), polypyrimidine tract-binding protein (PTB/hnRNPI) (siRNA-PTB), poly(C) binding protein 2 (PCBP2/hnRNP-E2) (siRNA-PCBP2), polyadenylate-binding protein-1 (PABP) (siRNA-PABP), eukaryotic translation initiation factor 4E (eIF4E) (siRNA-eIF4E), eukaryotic translation initiation factor 4E (eIF4G) (siRNA-eIF4G) or control siRNA (siRNA-control). Protein expression was determined by Western blotting using each specific antibody. GAPDH or tubulin was used as control. (A) La, (B) GAPDH, (C) PTB/hnRNPI, (D) PCBP2, (E) PABP, (F) eIF4E, and (G) eIF4G expressions are shown. (H) Effects of each siRNA on the HAV IRES activities. Huh7 cells were cotransfected with 0.3 µg pSV40-HAV-IRES [2] with each siRNA at 100 nM. Cells were harvested 48 h post-transfection and luciferase activities were measured. Activities of HAV IRES were calculated as previously described [17], [21]. Data are expressed as mean ± SD. *P<0.05 vs. Huh7 cells transfected with (siRNA-control).

Although the exact mechanisms are not fully understood, it has been reported that HAV IRES could interact with various endogenous genes [27]–[34], suggesting important roles of these proteins in HAV IRES-mediated translation and HAV replication. La, GAPDH, PTB and PCBP2 have been shown to bind to HAV IRES domains IIIb and V [23], IIIa [28], I-IIIb [30] and I-IIIb [32], respectively. PABP, eIF4E and eIF4G also interact with HAV IRES [33]–[36]. To determine whether each siRNA against these factors had a specific siRNA effect, knockdown of these molecules was validated by Western blotting, respectively (Figure 1A–1G). To examine the effects of the knockdown of these genes on HAV IRES-mediated translation, Huh7 cells were cotransfected with each siRNA, and pSV40-HAV-IRES, which contains SV40 promoter, renilla luciferase (Rluc) and nt. 139–854 of HAV sequence fused firefly luciferase (Fluc) gene [2]. After 48 h transfection, the cell lysates were analyzed for HAV IRES activities (Fluc/Rluc) as previously described (Figure 1H) [17], [21]. Compared with the HAV IRES activity in Huh7 cells transfected with control siRNA (siRNA-control) (100%), that transfected with siRNA-La was 39%, but those of the others were not inhibited (Figure 1H). These results provide further evidence of La being a potential cofactor for HAV IRES activity, indicating the possible usefulness of siRNA-La against HAV infection.

### Effects of silencing of La on HAV subgenomic replication

**Figure 2:**
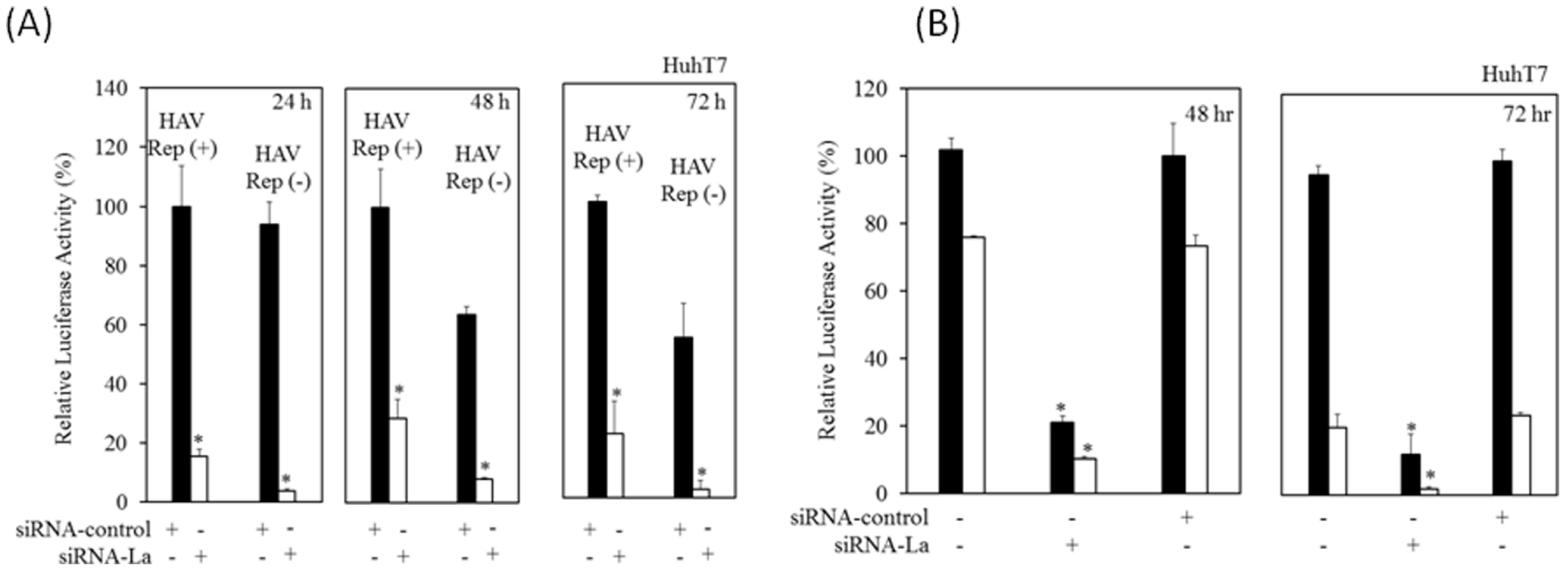
Knockdown of La inhibits hepatitis A virus (HAV) subgenomic replication. (A) Effects of siRNA against La (siRNA-La) on the HAV replication-competent replicon pT7-18f-LUC [HAV Rep (+)] or replication-incompetent replicon pT7-18f-LUCmut [HAV Rep (−)] replication. Approximately 0.5×10^6^ HuhT7 cells were cotransfected with 0.3 µg pT7-18f-LUC/pT7-18f-LUCmut [42] and 100 nM siRNA against La (siRNA-La)/control siRNA (siRNA-control). After 24 h (left), 48 h (middle) or 72 h (right), cell lysates were collected and luciferase activities were measured. (B) Effects of siRNA against La (siRNA-La) with or without amantadine on the HAV subgenomic replication-competent replicon pT7-18f-LUC. Approximately 0.5×10^6^ HuhT7 cells were cotransfected with 0.3 µg pT7-18f-LUC and 50 nM siRNA against La (siRNA-La)/control siRNA (siRNA-control). After 24 h transfection, cells were treated with 5 µg/mL amantadine (white column) or without (black column). After 48 h (left) or 72 h (right) transfection, cell lysates were collected and luciferase activities were measured. Data are expressed as mean ± SD. *P<0.05.

Next, we examined the effect of the silencing of La on HAV subgenomic replication [42] (Figure 2). To test luciferase activity due to translation or translation and replication, we introduced a replication-competent HAV replicon (pT7-18f-LUC) and a replication-incompetent HAV replicon (pT7-18f-LUC mut) into HuhT7 cells [21], [42], with or without amantadine treatment, which is effective for suppressing HAV replication [2], [21]. Reporter assays were performed 24 h, 48 h or 72 h after transfection. Relative luciferase activities of pT7-18f-LUC cotransfected with siRNA-control or siRNA-La, respectively, were 100% or 15.4% at 24 h, 100% or 28.7% at 48 h, and 100% or 21.7% at 72 h after transfection (Figure 2A). On the other hand, those of pT7-18f-LUC mut cotransfected with siRNA-control or siRNA-La, respectively, were 94% or 3.7% at 24 h, 63.8% or 7.9% at 48 h, and 54.2% or 2.9% at 72 h after transfection (Figure 2A). Because the luciferase values of pT7-18f-LUC or pT7-18f-LUC mut were due to translation with replication or translation without replication, respectively [21], [42], it was confirmed that siRNA-La might suppress HAV IRES-mediated translation. The effects of siRNA-La also enhanced the amantadine induced-suppression of HAV subgenomic replication (Figure 2B).

### JAK inhibitors AG490 and SD-1029 could suppress La expression

**Figure 3:**
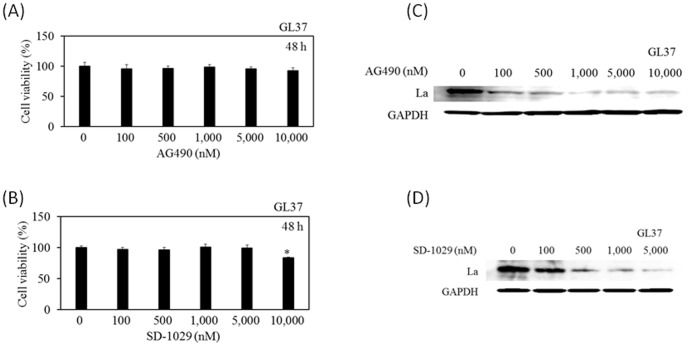
Effects of AG490 and SD-1029 on cell viability and La expression in GL37 cells. MTS assays of cells 48(A) or SD-1029 (B) at indicated concentrations. Data are expressed as mean ± SD. Western blotting analysis. Approximately 1×10^5^ cells were incubated in the presence of AG490 (C) or SD-1029 (D) at indicated concentrations. Twenty-four hours after treatment, cell lysates were analyzed for La and GAPDH expressions using specific antibodies. Data are expressed as mean ± SD. *P<0.05.

Because it was reported that La expression is dependent on JAK2^V617F^ in murine pro B Ba/F3-EPOR-derived cell line [42], the effects of two JAK2 inhibitors, AG490 and SD-1029, on La expression were examined. Initially, we evaluated the cytotoxicity of AG490 and SD-1029 on African green kidney GL37 cells by 3-(4,5-dimethylthiazol-2-yl)-5-(3-carboxymethoxyphenyl)-2-(4-sulfophenyl)-2H-tetrazolium, inner salt (MTS) assay. AG490 concentration in a range of 100–10,000 nM and SD-1029 concentration in a range of 100–5,000 nM were not toxic in 48-h incubation (Figure 3A, 3B). With these concentrations, we tested the effects of AG490 and SD-1029 on La expression in GL37 cells, which supports HAV replication [21]. The results of Western blotting showed that La expression was decreased in a concentration-dependent manner with AG490 (Figure 3C) and SD-1029 (Figure 3D). These data prompted us to examine whether these drugs had an inhibitory effect on HAV IRES-mediated translation or HAV replication.

### Effects of AG490 and SD-1029 on HAV IRES-mediated translation

**Figure 4:**
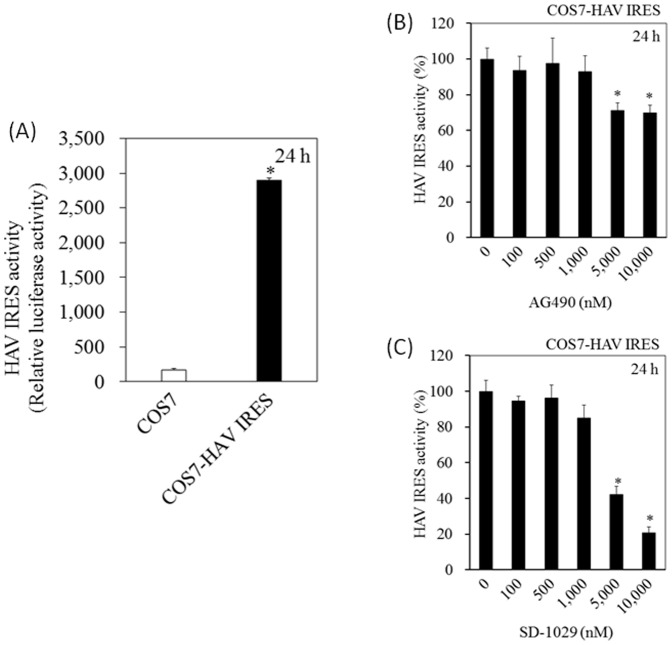
Effects of AG490 and SD-1029 on hepatitis A virus (HAV) internal ribosomal entry site (IRES) activities. (A) HAV IRES activities of COS7 cells stably expressing pSV40-HAV-IRES (COS7-HAV IRES cells). 0.5×10^5^ cells were seeded, cell lysates were collected 24 h later, and luciferase assay was performed for determination of HAV IRES activities. (B) Effect of AG490 on HAV IRES activity in COS7-HAV IRES cells. (C) Effect of SD-1029 on HAV IRES activity in COS7-HAV IRES cells. The cells were cultured with AG490 or SD-1029 at the concentrations indicated, and reporter assay was performed after 24 h of treatment. Activities of HAV IRES were calculated as previously described [17], [21]. Data are expressed as mean ± SD. *P<0.05.

COS7 cells stably expressing pSV40-HAV-IRES (COS7-HAV-IRES cells) were generated. To evaluate HAV IRES activity, after COS7 cells were cotransfected with pSV40-HAV IRES and pCXN2, and cultured in the presence of 500 µg/mL G418 for 3 weeks, COS7-HAV-IRES cells were established, making it easy to evaluate HAV IRES activity (Figure 4A). Treatment of these cells with AG490 resulted in the inhibition of HAV IRES activities (100%, 94%, 99%, 93%, 71% and 70% at 0, 100, 500, 1,000, 5,000 and 10,000 nM AG490, respectively) (Figure 4B). Treatment of these cells with SD-1029 resulted in the inhibition of HAV IRES activities (100%, 95%, 96%, 85%, 42% and 21% at 0, 100, 500, 1,000, 5,000 and 10,000 nM SD-1029, respectively) (Figure 4C).

### Effects of AG490 and SD-1029 on HAV replication

**Figure 5:**
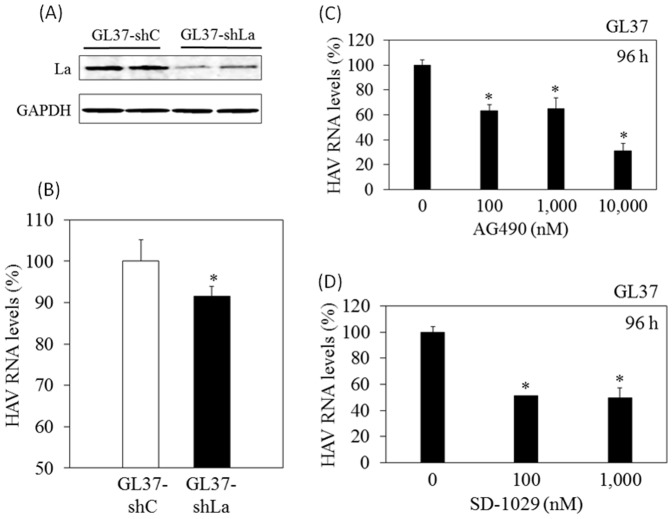
Antiviral activities of shRNA-La, AG490 and SD-1029 on hepatitis A virus (HAV) replication. (A) La protein expression in GL37 stably expressing shRNA-La (GL37-shLa cells) and GL37 stably expressing control shRNA (GL37-shC cells). Western blotting was performed with specific antibodies indicated. (B) Real-time PCR analysis of intracellular HAV RNA following HAV HA11-1299 genotype IIIA strain infection in GL37-shC or GL37-shLa cells. (C) Suppression of HAV infection in GL37 treated with AG490 at concentrations indicated. (D) Suppression of HAV infection in GL37 treated with SD-1029 at concentrations indicated. Cells were treated with AG490 or SD-1209 for 24 h, infected with HAV HA11-1299 genotype IIIA strain at MOI of 0.1, and washed with PBS 7 h later. After 96 h of HAV infection, cellular RNA was extracted, and HAV RNA levels were determined using real-time RT-PCR. Data are expressed as mean ± SD. *P<0.05.

We established GL37 stably expressing both short hairpin (sh)RNA-La (GL37-shLa cells) and control shRNA (GL37-shC cells) after GL37 cells were cotransfected with plasmid shRNA-La and plasmid shRNA-control, respectively, and cultured in the presence of puromycin. We examined whether shRNA-La could inhibit the replication of HAV HA11-1299 genotype IIIA strain in these GL37-derived cell lines. Western blotting analysis demonstrated that knockdown of La was validated in GL37-shLa cells, compared to GL37-shC cells (Figure 5A). As shown in Figure 5B, HAV RNA levels were 6.07×10^5^ copies/µg cellular RNA (92%) in GL37-shLa cells, in comparison with 6.63×10^5^ copies/µg cellular RNA (100%) in GL37-shC cells after 72 h of HAV infection at a multiplicity of infection (MOI) of 0.1.

Next, we investigated whether AG490 or SD-1029 could inhibit the replication of HAV HA11-1299 genotype IIIA strain in GL37 cells. Cells were treated with AG490 or SD-1029 for 24 h, infected with HAV HA11-1299 genotype IIIA strain at MOI of 0.1, and washed with PBS 7 h later. After 96 h of HAV infection, cellular RNA was extracted, and HAV RNA levels were determined using real-time RT-PCR. As shown in Figure 5C, HAV RNA levels were 5.27×10^4^, 5.46×10^4^ or 2.58×10^4^ copies/µg of cellular RNA (63%, 65% or 31%) in GL37 treated with 100, 1,000 or 10,000 nM AG490, respectively, in comparison with 8.35×10^4^ copies/µg of cellular RNA (100%) in GL37 without any treatment after 96 h of HAV infection at MOI of 0.1. As shown in Figure 5D, HAV RNA levels were 4.26×10^4^ or 4.12×10^4^ copies/µg cellular RNA (51% or 49%) in GL37 treated with 100 or 1,000 nM SD-1029, respectively, in comparison with that in GL37 without any treatment after 96 h of HAV infection at a MOI of 0.1. ELISA analysis of tissue culture-adapted HAV KRM003 genotype IIIB strain in GL37 cells [21] also showed mild inhibition of viral propagation with 500–1,000 nM AG490 but not with SD-1029 at 48 h post-infection (data not shown).

## Discussion

In the present study, we examined the effects of the knockdown of La in cell lines infected with HAV. We observed the inhibition of HAV replication by sh-La. We also observed that inhibitors of La, AG490 and SD-1029, induced the suppression of HAV genotype IIIA replication. Of course, HAV vaccine has already been developed. Although patients with acute hepatitis A are not usually treated with antiviral drugs, there are occasionally patients with severe acute hepatitis A such as fatal acute liver failure. To our knowledge, ours is the first study to report that a reduction of La can suppress HAV replication in cell culture.

It has been reported that down-regulation of La was induced by (-)-epigallocatechin gallate, iron chelator deferxamine and JAK inhibitor AZD1480 [43]–[45]. Ferric ammonium citrate up-regulates La expression [45]. It was reported that HBSC-11, an inhibitor of La, has an anti-HBV activity in which HBSC-11 may be mediated by a reduction in La levels [46]. Because we did not observe any effects of (-)-epigallocatechin gallate or ferric ammonium citrate on La expression in our experiments (data not shown), we chose JAK inhibitors in the present study. Our study suggested that anti-HAV activity of AG490 and SD-1029 should also be mediated by a reduction of La. It is possible that La inhibitors could be useful as antiviral drugs.

The use of 1,000 nM of AG490 and SD-1029 reduced La expression in GL37 cells (Figure 3C, 3D). However, there were no effects on HAV IRES activities up to this concentration in COS7-HAV-IRES cells (Figure 4B, 4C). These discrepancies might be a result of these two different cell lines, or this might be one of the points needing improvement in COS7-HAV-IRES cells.

Although AZD1480 was an inhibitor of JAK1 and JAK2 [46], AG490 is a tyrosine kinase inhibitor of JAK2, JAK3, epidermal growth factor (EGFR) and v-erb-b2 avian erythroblastic leukemia viral oncogene homolog 2 (Neu) [47], [48], and JAK2 inhibitor III SD-1029 acts as a JAK2-selective inhibitor [49]. Of interest is that these three JAK inhibitors reduce cellular La expression (Figure 3C and 3D) [45]. HAV and HCV modulate the JAK/STAT signaling pathway [50], [51]. Further studies will be needed at this stage, although several specific JAK inhibitors have been developed and there are ongoing trials for the treatment of myeloproliferative neoplasms [52], allergic skin diseases [53] and rheumatoid arthritis [54].

HAV replicates in the cytoplasm of hepatocytes, although La exists predominantly in the nucleus [37]. Previous studies have suggested that La associates with IRES-mediated translation [27], [40], [41], and the present study also demonstrated that La plays a potential role in HAV IRES-mediated translation. Our result also showed that HAV IRES-mediated translation was helped by La, in contrast to the previous observation [27]. These differences might be related to the experimental system such as in vivo or in vitro, and cell lines. Although HAV RNA levels were reduced by 5.6×10^4^ copies/µg cellular RNA when comparing GL37-shLa cells with GL37-shC cells (Figure 5B), it might be possible that other host factors are involved in suppressing HAV replication by JAK inhibitors. Further studies will be needed.

In the study field of HCV infection, the development of two distinct antiviral agents, DAAs and HTAs, could lead to higher sustained virological response rates via reductions of adverse events and treatment duration [9] compared to the former standard treatment [55]. The use of La inhibitor, one of the HTAs for HAV, alone or in combination with DAAs, might be beneficial for certain patients infected with HAV.

There are three HAV genotypes, I, II and III, of human origin [56]. The inhibitory effects of AG490 and SD-1029 on HAV subgenotype IIIA strain were observed by real-time PCR methods. But only weak inhibition of AG490 on HAV subgenotype IIIB was observed by ELISA methods. This may be related to the different methods of detection for HAV, that is, by RT-PCR or ELISA. There might also be differences among the different HAV subgenotypes, although HTAs have a high genetic barrier to resistance and a pan-genotypic antiviral activity [57]. Further studies on the exact mechanism of the association between La and HAV replication will be needed. In conclusion, inhibition of La by siRNAs and chemical agents could lead to the inhibition of HAV IRES-mediated translation and HAV replication in cell culture models. Our findings suggest that La plays important roles in HAV replication and should be exploited as one of the therapeutic targets.

## Materials and Methods

### Cells, virus and reagents

Human hepatoma cells (Huh7 and HuhT7, which stably express T7-RNA polymerase [42]) and African green monkey kidney cells (COS7 and GL37 [21], [22], [56], [58]), were grown in Dulbecco's modified essential medium (DMEM, Sigma-Aldrich, St. Louis, MO, USA) containing 10% fetal bovine serum, 100 units/ml penicillin and 100 µg/ml streptomycin (Sigma) at 37°C in 5% CO_2_. Huh7 and COS7 cells were purchased from JCRB cell bank, National Institute of Biomedical Innovation, Osaka, Japan. HAV subgenomic replicon was previously described [42]. Briefly, the structures of the competent HAV replicon (HAV) and incompetent HAV replicon (mut-HAV replicon) containing a frame-shift mutation in polymerase 3D were reported [42]. These replicons also contain an open-reading frame of firefly luciferase. HAV HA11-1299 genotype IIIA strain and HAV KRM003 genotype IIIB strain [21] were also used. The simian virus (SV) 40 promoter plasmid pSV40-HAV IRES was used as reported previously [2], [17]; it encodes in a bicistronic fashion the Renilla reniformis luciferase (Rluc), the hepatitis A virus internal ribosomal entry site (HAV IRES), followed by the firefly luciferase (Fluc). AG490 (Calbiochem, Billerica, MA, USA), SD-1029 (Santa Cruz Biotechnology, Santa Cruz, CA, USA) and amantadine (Sigma) were used in the present study.

### Transfection of shRNA and siRNAs

To stably establish GL37-shLa and control GL37-shC cells, respectively, we used the plasmids shRNA-La (shLa) and shRNA-control (shC) purchased from Santa Cruz. After electroporation of the plasmids, GL37 cells were placed in 10-mm-well plates (Iwaki Glass, Tokyo, Japan), and treated with 3 µg/mL puromycin for selection of antibiotic-resistant colonies over a 2-week period. We then confirmed the expression of endogenous La by Western blotting. siRNAs against La (siRNA-La), GAPDH (siRNA-GAPDH), PTB (siRNA-PTB), PCBP2 (siRNA-PCBP2), PABP (siRNA-PABP), eIF4E (siRNA-eIF4E), eIF4G (siRNA-eIF4G) and control siRNA (siRNA-control) were purchased from Santa Cruz.

### Transfection and luciferase assay

Huh7 cells were seeded in 6-well plates one day before transfecton, and cotransfected with 0.3 µg of the pSV40-HAV-IRES plasmid and 100 nM of each siRNA using Effectene transfection reagent (Qiagen, Hilden, Germany). Forty-eight hours after transfection, cell lysates were collected using a luciferase lysis buffer (Promega, Madison, WI, USA) according to the manufacturer's instructions. Luciferase activity was measured with a luminometer (AB-2200-R; ATTO, Tokyo, Japan).

### Western blotting

Cells were lysed in sodium dodecyl sulfate sample buffer, and after sonication, lysates were used for Western blotting analysis. Briefly, proteins were subjected to electrophoresis on 5–20% polyacrylamide gels and transferred onto polyvinylidene difluoride membranes (ATTO, Tokyo, Japan). Membranes were incubated with specific antibodies for La, GAPDH, PTB, PCBP2, PABP, eIF4E, eIF4G and tubulin (Santa Cruz). After washing, membranes were incubated with secondary horse-radish peroxidase–conjugated antibodies. Signals were detected by means of enhanced chemiluminescence (GE Healthcare, Tokyo, Japan) and scanned by image analyzer LAS-4000 and Image Gauge (version 3.1) (Fuji Film, Tokyo, Japan) and Scion Image (Scion) software.

### RNA extraction and real-time RT-PCR

After 96 h or 72 h of HAV infection, total RNA was isolated using the RNeasy Mini Kit (Qiagen). One microgram of RNA was reverse-transcribed with the PrimeScript RT reagent (Perfect Real Time; Takara, Otsu, Japan). PCR amplification was performed on cDNA templates using primers specific for HAV (sense primer 5′-AGGCTACGGGTGAAACCTCTTAG-3′ and antisense primer 5′-GCCGCTGTTACCCTATCCAA-3′) [59]. For RNA quantification, real-time PCR was performed using Power SYBR Green Master Mix (Applied Biosystems, Forester City, CA, USA) following the manufacturer's protocol. Data analysis was based on the Standard curve method.

### MTS assay

To evaluate cell viability, MTS assays were performed using a Cell Titer Aqueous One Solution Proliferation Assay (Promega) according to the manufacturer's instructions.

### Statistical analysis

Statistical analysis was performed using Student's t-test. *P*-values <0.05 were considered statistically significant.
